# Dietary Cholesterol Differentially Regulates the Muscle Lipidomics of Farmed Turbot and Tiger Puffer

**DOI:** 10.3390/ani13101632

**Published:** 2023-05-13

**Authors:** Xiaoxue Meng, Qingzhu Bi, Qiang Ma, Yuliang Wei, Yanlu Li, Mengqing Liang, Houguo Xu

**Affiliations:** 1 Yellow Sea Fisheries Research Institute, Chinese Academy of Fishery Sciences, 106 Nanjing Road, Qingdao 266071, China; mengxx127@163.com (X.M.);; 2 College of Fisheries, Guangdong Ocean University, 1 Haida Road, Zhanjiang 524008, China

**Keywords:** flatfish, pufferfish, cholesterol, muscle, lipidomics

## Abstract

**Simple Summary:**

In some fish farming practices, cholesterol has been used as a feed additive for growth stimulation. Therefore, it is important to increase our knowledge and experience about dietary cholesterol effects on fish. This study provides useful information for the application of cholesterol in fish diets by analyzing the muscle lipidomics changes in turbot and tiger puffer in response to dietary cholesterol supplementation.

**Abstract:**

Exogenous cholesterol has been supplemented into aqua-feeds due to the reduced proportions of fishmeal and fish oil. This study aimed to investigate the effects of dietary cholesterol supplementation on the muscle lipidomics of two marine fish species, turbot and tiger puffer. A 70-day feeding trial was conducted, where two low-fishmeal diets supplemented with 0 or 1% cholesterol were used. The lipidomic analysis with targeted tandem mass spectrometry showed that, in turbot, a total of 49 individual lipids exhibited significant differences in their abundance in response to dietary cholesterol, whereas the number was 30 for tiger puffer. Dietary cholesterol up-regulated the abundance of cholesterol and cholesterol ester in both species. In turbot, the dietary cholesterol also increased the abundance of triacylglycerol and acylcarnitine, whereas in tiger puffer, it primarily regulated the abundance of phospholipids and BMP. This was the first time the responses of marine fish muscle lipidomics to dietary cholesterol supplementation have been investigated.

## 1. Introduction

Cholesterol is a very important steroid for animals [1,2,3]. For fish, cholesterol does not appear to be an indispensable nutrient in their feed, because it can be bio-synthesized. Additionally, the traditional ingredients of fish feed, fishmeal, and fish oil, are rich in cholesterol. However, in modern fish feeds, the inclusion levels of fishmeal and fish oil are rapidly decreasing due to the booming aquaculture industry.

In diets with low fishmeal and fish oil levels, whether cholesterol supplementation is necessary has been controversial across fish studies [4]. Some studies have shown that dietary cholesterol supplementation could significantly increase the growth performance of fish [5,6,7,8,9,10,11], whereas opposite results have been reported in other studies [6,12,13,14,15]. As already mentioned in our previous studies [4], the cholesterol requirement of most marine carnivorous fish is around 1% and the protein source may have a great impact on the cholesterol requirements of fish. Although some terrestrially sourced oils, such as beef tallow, lard, and poultry oil, can also provide certain levels of cholesterol, these oils are still not commonly used in fish feed. In some fish farming practices, cholesterol has been used as a feed additive for growth stimulation. A dietary supplementation of cholesterol has been assumed to be able to spare the energy used for endogenic cholesterol bio-synthesis. 

A recent study with turbot and tiger puffer revealed that cholesterol supplementation did not significantly affect the fish growth performances (but 1% cholesterol supplementation resulted in the best growth performance in tiger puffer), whereas it significantly regulated their lipid accumulation and metabolism [4]. Regarding the effects of dietary cholesterol on fish muscle lipid accumulation, different results have been reported for other fish species, such as hybrid striped bass (*Morone chrysops* × *M. saxatilis*) and rainbow trout (*Oncorhynchus mykiss*) [9,10,13]. In mammalian studies, links between cholesterol concentration and muscle contractile force have been found [16,17]. Another recent study reported that dietary cholesterol supplementation regulated the fillet pigmentation of female triploid Atlantic salmon (*Salmo salar*) reared in high temperatures [18]. To date, the effects of cholesterol supplementation in fish feed on fish muscle lipid profiles are not well known. Due to their detailed genome information and special lipid storage patterns, turbot (*Scophthalmus maximus*) and tiger puffer (*Takifugu rubripes*) are becoming good research models [19]. With samples from a previous feeding trial [4], the current study aimed to evaluate the effects of cholesterol supplementation on the muscle lipidomics of juvenile turbot and tiger puffer, both of which are also important aquaculture species. 

## 2. Materials and Methods

### 2.1. Diets, Fish Rearing and Sampling

Muscle samples were collected from a recent feeding trial. The detailed methods for the diet preparation, fish rearing, and sampling have previously been described in detail [4]. A basal diet (30% fishmeal, no fish oil) without cholesterol supplementation was used as the control diet (Table S1 and Table 1). Pure cholesterol (purity ˃ 99%, Macklin) was added into the control diet at 1.0% to obtain the treatment diet (Diet CHO). The cholesterol concentrations in the control and CHO diets were measured to be 0.11% and 1.10%, respectively. 

The average initial weights of the turbot (*Scophthalmus maximus*) and tiger puffer (*Takifugu rubripes*) used in the current study were 21 and 12 g, respectively. The fish were reared in indoor tanks (200 L, 30 fish/tank, 3 tanks/diet). The turbot and tiger puffer were fed to apparent satiation at different frequencies (turbot, 7:00 and 19:00; tiger puffer, 6:30, 12:30, and 18:30) due to the intrinsic differences in the digestive characteristics between the two fish species. A flow-through seawater system was used. The feeding duration was 70 days. During the experiment, the water temperature ranged from 19 to 21 °C, the salinity from 28 to 30, the pH from 7.6 to 7.9, and the dissolved oxygen was >8 mg L^−1^.

At the end of the feeding experiment, the sampling was conducted after a 24 h fasting of the fish. At this time, the average final weights of the turbot in the control and CHO groups were 74.08 g and 73.76 g, respectively, and those of the tiger puffer were 43.27 g and 45.69 g, respectively. The muscle samples of six randomly selected fish from each tank were obtained and pooled as one sample for the analyses of the crude lipid content, fatty acid composition, and lipidomics. All the fish-rearing practices and sampling protocols in this study were approved by the Animal Care and Use Committee of Yellow Sea Fisheries Research Institute.

### 2.2. Muscle Lipidomic Assay

The detailed method for the targeted lipidomic analysis is available in a recent publication [20]. Briefly, the total lipid was extracted with the chloroform: methanol method according to Lam et al. [21], with modifications. A tailored, extensive, and targeted lipid library was used in the lipidomics analysis. This lipid library confers sufficient coverage for a global lipid pathway analysis. Internal standard calibrations were used for all the lipid quantitations. The polar lipids were assayed with an Agilent 1290 UPLC system equipped with a triple quadrupole/ion trap mass spectrometer (6500 Plus Qtrap; SCIEX, [21]). The different individual polar lipid classes were separated with normal phase (NP)-HPLC using a silica column (Phenomenex Luna 3 µm, internal diameter 150 × 2.0 mm). The mobile phases A and B were “chloroform: methanol: ammonium hydroxide=89.5:10:0.5” and “chloroform: methanol: ammonium hydroxide: water = 55:39:0.5:5.5”, respectively. A series of internal standards were used for the quantitation of the same lipid classes [20]. 

Glycerol lipids were quantified with an HPLC/MRM method (reverse phase). Neutral lipid was separated with a 2.6 µm column (Phenomenex Kinetex-C18, 4.6 × 100 mm). The isocratic mobile phase contained chloroform: methanol:0.1 M ammonium acetate (*v*/*v*/*v* = 100:100:4, at a flow rate of 170 µL for 17 min). The spiked internal standards, TAG(14:0)_3_-d_5_, TAG(16:0)_3_-d_5_, and TAG(18:0)_3_-d_5_ (CDN isotopes, Pointe-Claire, Canada), were used for a quantitation of the triacylglycerols (TAG). The standards d_5_-DAG18:1/18:1 and d_5_-DAG17:0/17:0 (Avanti Polar Lipids, Alabaster, AL, USA) were used for a quantitation of the diacylglycerols (DAG). The internal standards d_6_-C18:0 cholesterol esters (CE) and d_6_-cholesterol (CDN isotopes, Pointe-Claire, Canada) were used for a quantitation of the CE and free cholesterols, respectively. For the quantitation of the free fatty acids, d_8_-20:4 (Cayman Chemicals, Ann Arbor, MI, USA) and d_31_-16:0 (Sigma-Aldrich (China) Shanghai, China) were used as internal standards, while the quantitation of the acyl-carnitines used d_3_-16:0-acylcarnitine (Cayman Chemicals, Ann Arbor, MI, USA) as an internal standard. The lipid content was expressed as μmol g^−1^ sample.

### 2.3. Total Lipid Content and Fatty Acid Profile

The muscle total lipid content was extracted using the chloroform–methanol method. The fatty acid composition was assayed using a gas chromatograph method (Shimadzu GC-2010 Pro), as previously described [4]. A flame ionization detector and fused silica capillary column (SH-RT-2560, 100 m × 0.25 mm × 0.20 μm, Shimadzu, Japan; dicyano-propyl-polysiloxane as stationary phase) were used. The results are expressed as the percentages of each fatty acid with respect to the total fatty acids.

### 2.4. Statistics

For the lipidomics data, the relative contents of the individual lipids between the two groups were a focus in the results description. The Games-Howell test was applied in a parametric test on the inter-group difference. A two-sided *p* < 0.05 was considered as statistically significant. A principal component analysis (PCA) analysis was performed with FactoMineR (V2.4). The heatmap plot was created using the ComplexHeatmap (V2.10.0) package. The Ggplot2 (V3.3.3) package and R 4.0 software were used when other plots, such as boxplot and barplots, were created. 

For the lipid content and fatty acid composition data, the statistical analyses used T Tests for the independent samples in SPSS 16.0 (the percentage data were firstly arcsine transformed). Significant differences were considered when *p* < 0.05.

## 3. Results and Discussion

A total of 627 individual lipid metabolites were quantified in this study (Supplementary Material, raw data, and Figure S1). The largest lipid class was TAG (130 species), followed by cardiolipin (CL) (59 lipid species), phosphatidylcholine (PC) (48 lipids), and phosphatidylethanolamine (PE) (46 lipids). A total of 10–30 individual lipids were quantified in most of the other lipid classes identified, while only 1 lipid species was identified and quantified for bis(monoacylglycerol)phosphate (BMP), sphingosine (Sph), and ceramide trihexoside (Gb3).

**Figure 1:**
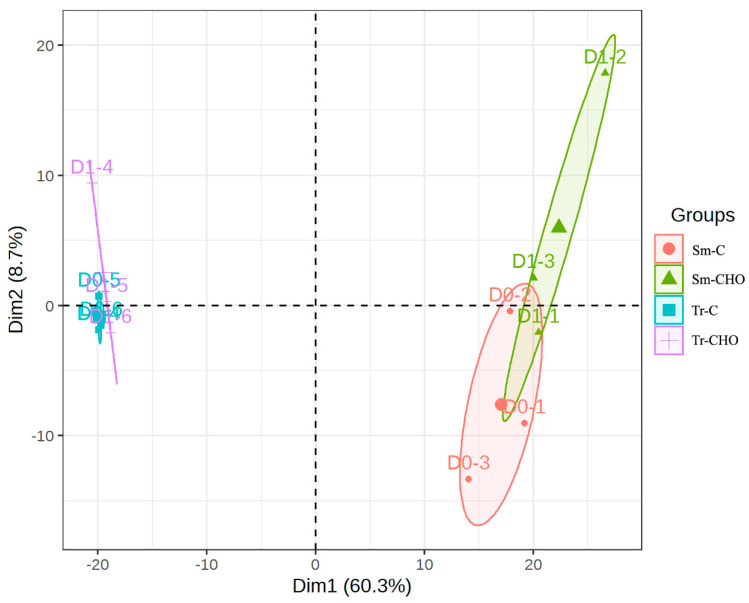
The principal component analysis (PCA) of the lipidomics. Sm: *Scophthalmus maximus* (turbot); Tr: *Takifugu rubripes* (tiger puffer); C: the control group; and CHO: the cholesterol−supplemented group.

For both fish species, the PCA showed that the control and CHO groups clustered quite separately (Figure 1, Figures S2 and S3). However, compared to the turbot, the distance between the two groups of the tiger puffer was shorter. This was consistent with the result that the turbot had more lipids (49), showing inter-group differences in abundance in comparison to the tiger puffer (30) (Table 2). The abundance of most of these lipids (35 for the turbot and 22 for the tiger puffer) was up-regulated by the cholesterol supplementation. These results are also consistent with the fact observed in our previous studies, which was that tiger puffer had a higher buffering capacity than turbot when responding to a change in the cholesterol level in the diet [4]. 

**Figure 2:**
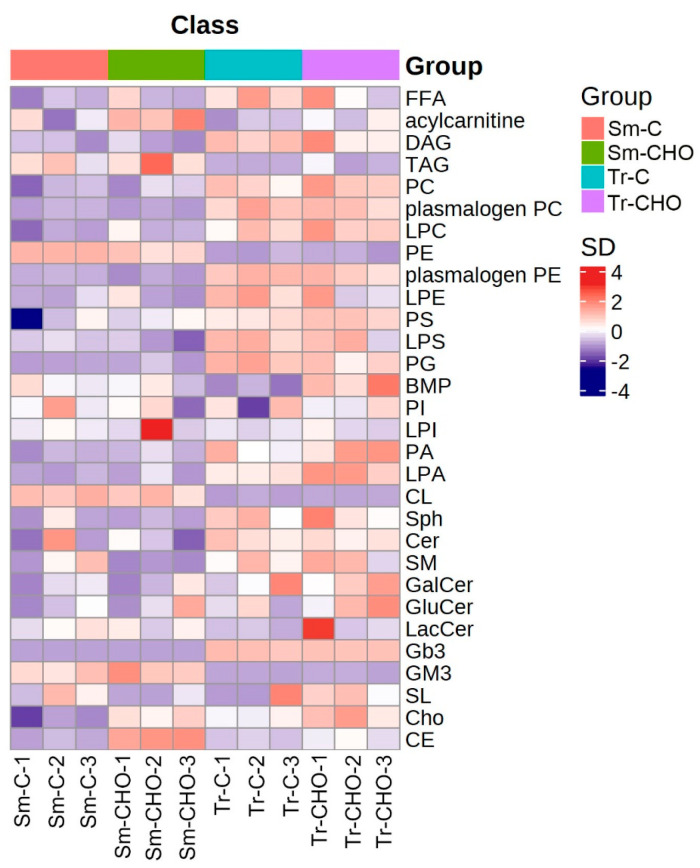
Heatmap of quantified lipid classes. The average concentration of a lipid class was standardized to be 0. Higher concentration than the average was labeled as orange, and lower concentration was labeled as purple. The color value as indicated in the right color bar means fold of standard deviation distant to the average concentration. Sm: *Scophthalmus maximus* (turbot); Tr: *Takifugu rubripes* (tiger puffer); C: the control group; and CHO: the cholesterol−supplemented group.

**Figure 3:**
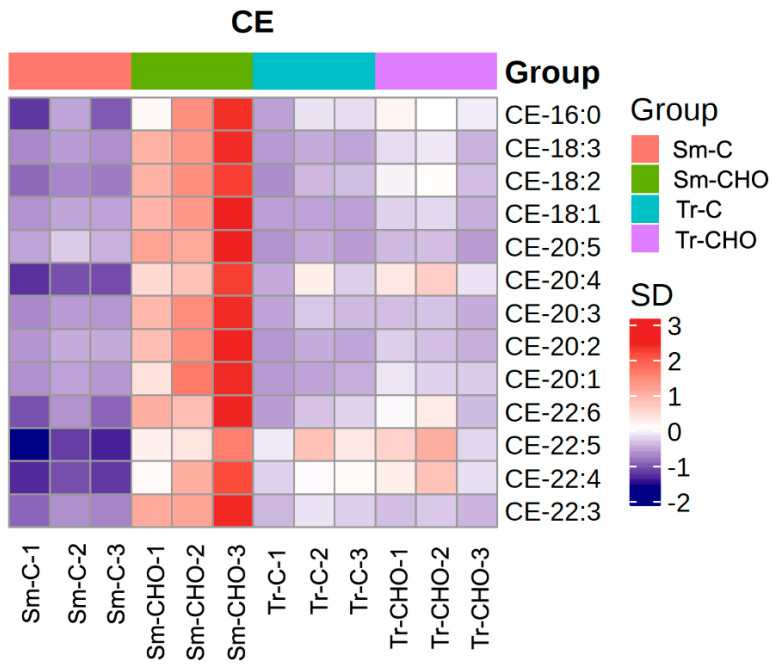
Heatmap of quantified cholesterol ester (CE). The average concentration of a lipid metabolite was standardized to be 0. Higher concentration than the average was labeled as orange, and lower concentration was labeled as purple. The color value as indicated in the right color bar means fold of standard deviation distant to the average concentration. Sm: *Scophthalmus maximus* (turbot); Tr: *Takifugu rubripes* (tiger puffer); C: the control group; and CHO: the cholesterol−supplemented group.

Regarding the specific lipid composition changes caused by dietary cholesterol supplementation, both similar and different trends were observed for these two fish species. Firstly, in both species, the total lipid content (Figure S4) and fatty acid composition (Table 1) in the muscle were not significantly affected by dietary cholesterol supplementation. From the lipidomics results, as expected, the most significantly affected lipid classes in the muscle were cholesterol and CE (Figure 2 and Figure 3), as similarly observed in rainbow trout [9,10]. However, in the turbot, the abundance of the lipid class CE in the CHO group was 6.23 times that of the control group, whereas it only changed by 1.55 folds in the tiger puffer (Table 2 and Table S2). The specific CEs significantly affected in the turbot muscle included CE-20:3 (Fold_CHO/control_ = 14.5), CE-18:2 (Fold_CHO/control_ = 7.69), CE-22:3 (Fold_CHO/control_ = 7.59), and CE-22:5 (Fold_CHO/control_ = 3.85), whereas the significantly affected CE in the tiger puffer muscle was CE-20:1 (Fold_CHO/control_ = 3.06). Considering that both the total and free fatty acid compositions were barely affected by the dietary cholesterol supplementation in this study, why these CE were affected by this dietary cholesterol supplementation remains unclear. In terrestrial animals, some CEs, such as CE-18:2, CE-18:1, CE-20:4, and CE-22:6, have been used as biomarkers for the early prediction of obesity and other clinical lesions [22,23,24]. However, in fish, no relevant information is available. Although in this study, dietary cholesterol supplementation did not significantly affect the muscle crude lipid level, in rainbow trout, a dietary cholesterol supplementation at 1.2–1.5% significantly increased the muscle crude lipid content [9]. 

One possible explanation for the difference in the change in the CEs between the two species is that turbot muscle may have a higher neutral lipid content than tiger puffer muscle. This hypothesis can also explain another major difference between the turbot and tiger puffer, that is, compared to the turbot, more phospholipids were affected by the dietary cholesterol supplementation in the tiger puffer. In the tiger puffer, 24 out of the 30 significantly affected lipid species were phospholipids, plasmalogen, or lysophospholipids, including PA38:3(18:0_20:3), PC40:6(22:5_18:1), PS38:6, PC36:2p(18:0p_18:2), PA40:6(18:0_22:6), LPA16:0, PA 36:1(18:0_18:1), LPI20:4, PC34:1(16:1_18:0), PC36:1p(18:0p_18:1), LPA16:1, PS38:3, PE40:5p(18:0p_22:5), PC34:3, PA38:4(18:0_20:4), PA40:5(18:0_22:5), PS38:4, PC32:1, LPI22:5, PA38:3, PE40:4p, PS38:3(18:0_20:3), PC34:2p(16:0p_18:2), and PC42:7(22:5_20:2), whereas in the turbot, less phospholipids were affected by the dietary cholesterol supplementation. In the turbot muscle, only 16 out of the 49 significantly affected lipid species were phospholipids, including PE36:1, PG38:4(18:0_20:4), PG36:4(16:0_20:4), PE38:5, PC42:5, PE38:5p, PE40:6(20:2_20:4), PC34:2p, PE36:3p(16:0p_20:3), PI38:5, PC38:2p, PG36:4(16:1_20:3), PS32:1, PE34:1, PC40:3p, and PC32:1. It was noteworthy that only two affected phospholipids, PC34:2p and PC32:1, were shared between the two fish species. These were also the only two affected individual lipids shared between the turbot and tiger puffer. Very little information is known about PC34:2p and PC32:1, except that PC32:1 has higher levels in newborns compared to 4-month old infants [25]. Regarding the inter-species differences in the responses of the muscle phospholipids to the dietary cholesterol supplementation, a major one was that the abundance of some PSs and PAs changed (mostly up-regulated) only in the tiger puffer (Figure S5). PS is an important constituent of cellular membranes, in particular in the nervous system [26]. Compared to turbot, which is a benthic flatfish, tiger puffer, as a fast-swimming species, may require a higher PS abundance in its body. PA is a well-established second messenger [27], and thus may play an important role in the metabolism regulation of dietary cholesterol supplementation. Additionally, as one of the major phospholipids in the plasma membrane, PA can be catalyzed by phospholipase A1/A2 to produce lysophosphatidic acid (LPA) [28], of which LPA16:0 and LPA16:1 in the tiger puffer were increased in abundance by the dietary cholesterol supplementation. Moreover, PA can be degraded by phospholipid hydrolases LPPs to produce DAG, which is the precursor substance of TAG [28]. Therefore, this increase in PA can be recognized as an early predictor of lipogenesis. A previous fish study showed that, after turbot muscle cells were stimulated with PA, the gene expression related to fatty acid synthesis was up-regulated, whereas the expression of the genes involved in fatty acid *β*-oxidation was down-regulated [29]. 

In contrast to the inter-species differences in the phospholipid changes, compared to the tiger puffer, more TAGs in the turbot were changed in abundance by the dietary cholesterol. Among the 49 significantly affected lipids in the turbot muscle, 13 were TAGs, with an average increase of 1.51 folds (Table 2, Figure S6). Both TAG and cholesterol are transported among tissues via lipoproteins, thus their contents may change concurrently. Dietary cholesterol may also affect the TAG concentration in indirect ways. Dietary cholesterol supplementation has been found to up-regulate the hepatic gene expression of SREBF1 [4], which is a key transcription factor in lipogenesis. Additionally, an increase in cholesterol uptake will increase the biosynthesis of bile acids [4] and an increased bile acid level may further regulate the lipid digestion and lipid/cholesterol transport in fish [30]. 

**Figure 4:**
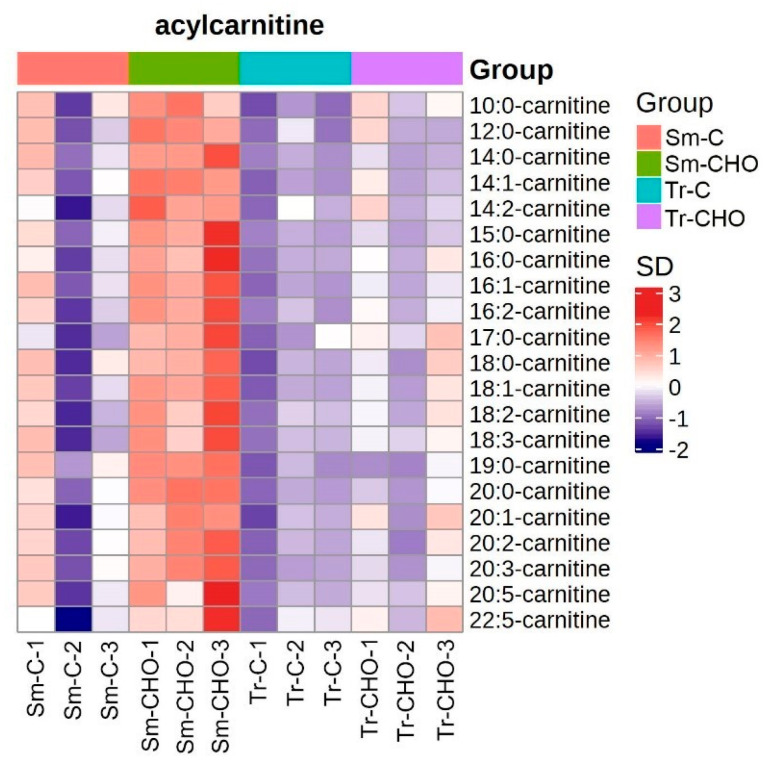
Heatmap of quantified acylcarnitines. The average concentration of a lipid metabolite was standardized to be 0. Higher concentration than the average was labeled as orange, and lower concentration was labeled as purple. The color value as indicated in the right color bar means fold of standard deviation distant to the average concentration. Sm: *Scophthalmus maximus* (turbot); Tr: *Takifugu rubripes* (tiger puffer); C: the control group; and CHO: the cholesterol−supplemented group.

With respect to the other lipids differentially affected by the dietary cholesterol supplementation between the two fish species, the abundance of acylcarnitine was significantly increased by the cholesterol in the turbot muscle, but was changed to a much lesser extent in the tiger puffer (Figure 4). Acylcarnitines are formed during the transport of long-chain fatty acids into the mitochondria [31] and are essential for energy metabolism and β-oxidation [32]. The increase in acylcarnitine was probably related to the stimulation of lipolysis [33]. However, in other studies, no direct link has been reported between dietary cholesterol and muscle acylcarnitine levels. 

In the tiger puffer muscle, the BMP (BMP 36:3 (18:2-18:1)) abundance was increased (Fold_CHO/control_ = 2.01) by the dietary cholesterol supplementation. As a negatively charged glycerophospholipid, BMP has an unusual sn-1:sn-1′structural configuration [34]. BMP has been demonstrated to be a key activator of cholesterol sorting and membrane digestion [35], and in pathology, it has been reported to be a peculiar phospholipid in controlling the fate of cholesterol [36]. In rat fibroblasts, BMP accumulation induced by the silencing of MPR was correlated with cholesterol accumulation [36]. It has also been proposed that BMP, in collaboration with Alix, regulates the cholesterol levels in late endosomes [37]. The present study showed similar results with the mammalian studies mentioned above regarding concurrent changes in BMP and cholesterol. However, the precise mechanisms involved in fish warrant further research. 

In the tiger puffer muscle, it was also noteworthy that the abundance of glucosylceramide (GluCer) d18:1/15:0 was increased (2.44 folds) by the dietary cholesterol supplementation (Table 2, Figure S7). In higher eukaryotes, GluCer is the simplest member and precursor of glycosphingolipids, which are organized in signaling domains on the cell surface and serve specialized functions in recognition processes [38]. The interplay between GluCer and cholesterol has been investigated in some in vitro studies, and concurrent increases in cholesterol and GluCer have been observed in some circumstances [39,40]. In fish, a study on Atlantic salmon showed that overfeeding with polyunsaturated fatty acids reduced the GluCer concentration in their serum, whereas overfeeding with saturated fatty acids increased it [41]. However, no previous research on fish has reported a direct link between GluCer and cholesterol. 

There were some other changes in the lipid profiles caused by the dietary cholesterol supplementation, but most of them were marginal and were thus not discussed in detail. In addition, alongside the previous growth results, the present results still indicate that the effects of dietary cholesterol supplementation on various fish physiological processes may not be synchronous with those on their growth performances. A more comprehensive evaluation should be conducted when cholesterol is added to fish feed as an additive. 

## 4. Conclusions

In conclusion, dietary cholesterol significantly altered the lipid compositions in the muscles of turbot and tiger puffer. It up-regulated the abundance of cholesterol and CEs in both fish species. Different effects were also observed between the two species. In the turbot, dietary cholesterol increased the abundance of TAG and acylcarnitine, whereas in the tiger puffer, it primarily regulated the abundance of BMP and phospholipids, in particular PS and PA. Further studies are needed to elucidate the precise mechanisms involved.

## Figures and Tables

**Table 1:** Fatty acid compositions of experimental diets and fish muscle (% total fatty acids, mean ± standard error).

Fatty Acid	Diet		Turbot Muscle		Tiger Puffer Muscle	
	Control	CHO	Control	CHO	Control	CHO
14:0	2.81	2.71	1.32 ± 0.09	1.19 ± 0.05	0.42 ± 0.01	0.45 ± 0.02
16:0	17.4	17.0	21.0 ± 0.14	21.7 ± 0.09 *	19.7 ± 0.14	20.3 ± 0.13 *
18:0	5.09	5.02	8.61 ± 0.02	8.13 ± 0.07	12.5 ± 0.08	12.1 ± 0.18
∑SFA	26.4	25.8	31.6 ± 0.13	31.7 ± 0.10	33.2 ± 0.18	33.5 ± 0.09
16:1n-7	3.23	3.26	1.49 ± 0.08	1.33 ± 0.02	0.65 ± 0.01	0.71 ± 0.03
18:1n-9	24.7	24.2	18.3 ± 0.40	17.5 ± 0.12 *	13.3 ± 0.13	13.9 ± 0.10 *
20:1n-9	0.62	0.60	0.56 ± 0.02	0.60 ± 0.03	0.78 ± 0.02	0.79 ± 0.01
∑MUFA	28.8	28.2	20.9 ± 0.51	20.0 ± 0.10 *	15.2 ± 0.16	16.0 ± 0.08 *
18:2n-6	23.3	23.4	19.3 ± 0.21	19.5 ± 0.21	21.3 ± 0.15	21.5 ± 0.22
20:2n-6	0.00	0.00	0.73 ± 0.05	0.79 ± 0.03	1.43 ± 0.03	1.31 ± 0.08
20:4n-6	0.45	0.45	1.15 ± 0.05	1.25 ± 0.01 *	1.34 ± 0.03	1.28 ± 0.04
∑n-6PUFA	24.0	24.1	21.2 ± 0.20	21.5 ± 0.22	24.1 ± 0.15	24.1 ± 0.25
18:3n-3	13.7	14.3	6.70 ± 0.14	6.51 ± 0.08	3.59 ± 0.10	3.72 ± 0.15
20:5n-3	4.32	4.67	6.32 ± 0.10	6.47 ± 0.10	5.21 ± 0.07	5.33 ± 0.06
22:5n-3	0.60	0.63	2.20 ± 0.01	2.27 ± 0.05	5.76 ± 0.09	5.01 ± 0.15 *
22:6n-3	2.13	2.28	10.5 ± 0.41	11.0 ± 0.23	12.1 ± 0.25	11.7 ± 0.42
∑n-3PUFA	20.8	21.9	26.2 ± 0.31	26.8 ± 0.39	27.5 ± 0.32	26.5 ± 0.29 *
n-3/n-6	0.87	0.91	1.24 ± 0.01	1.25 ± 0.03	1.14 ± 0.02	1.10 ± 0.02

* indicates significant (*p* < 0.05) difference between the two groups of turbot or tiger puffer. SFA: saturated fatty acids; MUFA: mono-unsaturated fatty acids; and PUFA: poly-unsaturated fatty acids. In addition to the fatty acids listed in the table, total fatty acids include 12:0, 13:0, 15:0, 17:0, 20:0, 21:0, 22:0, 14:1n-5, 15:1n-5, 17:1n-7, 22:1n-9, 18:3n-6, 20:3n-6, 22:2n-6, and 20:3n-3.

**Table 2:** Lipid metabolites in turbot and tiger puffer muscle having significantly (*p* < 0.05) different abundances between the cholesterol-supplemented (CHO) group and control group. Fold: CHO/control. “↑” and “↓” represents up- and down-regulation with dietary cholesterol supplementation, respectively.

Lipid	***p*** Value	Fold	Lipid	***p*** Value	Fold
Turbot			Tiger puffer		
*Class*			*Class*		
CE	0.0001	6.23↑	BMP	0.0203	2.01↑
Cholesterol	0.0127	1.26↑	PS	0.0273	1.03↑
*Specific lipid*			*Specific lipid*		
PE36:1	0.0011	0.81↓	PA38:3(18:0_20:3)	0.0006	1.26↑
PG38:4(18:0_20:4)	0.0058	1.31↑	CE-20:1	0.0084	3.06↑
TAG52:4(16:1)	0.0061	1.48↑	PC40:6(22:5_18:1)	0.0086	0.91↓
SM d18:1/23:0	0.0081	0.70↓	PS38:6	0.0087	1.13↑
PG36:4(16:0_20:4)	0.0090	1.36↑	PC36:2p(18:0p_18:2)	0.0099	1.13↑
TAG52:6(16:1)	0.0094	1.55↑	PA40:6(18:0_22:6)	0.0112	1.59↑
SM d18:1/22:1	0.0104	0.78↓	LPA16:0	0.0133	1.31↑
PE38:5	0.0123	0.88↓	PA 36:1(18:0_18:1)	0.0152	1.94↑
TAG52:6(18:3)	0.0125	1.52↑	LPI20:4	0.0175	0.78↓
PC42:5	0.0129	1.32↑	PC34:1(16:1_18:0)	0.0189	1.14↑
TAG52:5(16:0)	0.0133	1.51↑	PC36:1p(18:0p_18:1)	0.0195	1.07↑
CL72:5(18:2)	0.0148	0.90↓	BMP36:3(18:2_18:1)	0.0203	2.01↑
TAG56:7(22:5)	0.0166	1.65↑	LPA16:1	0.0205	1.26↑
17:0-carnitine	0.0194	2.31↑	PS38:3	0.0213	1.22↑
PE38:5p	0.0217	0.81↓	PE40:5p(18:0p_22:5)	0.0224	0.81↓
CE-18:2	0.0221	7.69↑	GluCer d18:1/15:0	0.0284	2.44↑
TAG52:5(16:1)	0.0235	1.49↑	PC34:3	0.0288	1.12↑
SM d18:1/22:0	0.0237	0.70↓	10:0-carnitine	0.0312	1.64↑
TAG52:4(16:0)	0.0239	1.38↑	PA38:4(18:0_20:4)	0.0316	1.51↑
PE40:6(20:2_20:4)	0.0249	1.21↑	PA40:5(18:0_22:5)	0.0369	1.39↑
PC34:2p	0.0263	0.83↓	PS38:4	0.0375	1.22↑
TAG48:4(16:1)	0.0297	1.82↑	PC32:1	0.0377	1.09↑
CE-22:3	0.0299	7.59↑	LPI22:5	0.0387	0.67↓
PE36:3p(16:0p_20:3)	0.0300	0.81↓	CL74:7(18:2)	0.0392	1.10↑
PI38:5	0.0302	0.85↓	PA38:3	0.0414	1.43↑
CE-22:5	0.0333	3.85↑	TAG58:9(22:5)	0.0432	0.77↓
PC38:2p	0.0337	0.86↓	PE40:4p	0.0470	0.85↓
CE-18:3	0.0340	10.9↑	PS38:3(18:0_20:3)	0.0479	0.90↓
PG36:4(16:1_20:3)	0.0346	1.24↑	PC34:2p(16:0p_18:2)	0.0484	1.15↑
TAG52:4(16:2)	0.0350	1.33↑	PC42:7(22:5_20:2)	0.0498	0.81↓
CE-20:3	0.0357	14.5↑			
PS32:1	0.0364	1.10↑			
TAG52:4(18:1)	0.0376	1.42↑			
CE-22:6	0.0377	3.44↑			
TAG52:5(18:3)	0.0382	1.48↑			
PE34:1	0.0396	0.82↓			
SM d18:1/14:1	0.0420	1.24↑			
CE-18:1	0.0428	14.9↑			
PC40:3p	0.0428	0.89↓			
CE-20:5	0.0432	5.23↑			
15:0-carnitine	0.0433	2.34↑			
PC32:1	0.0443	1.04↑			
CE-20:2	0.0467	14.4↑			
SM d18:1/23:1	0.0467	0.80↓			
14:2-carnitine	0.0486	1.97↑			
CE-20:4	0.0487	5.45↑			
TAG50:3(18:2)	0.0489	1.38↑			
TAG48:3(16:0)	0.0491	1.66↑			
16:0-carnitine	0.0497	2.45↑			

## Data Availability

The data presented in this study are available in the manuscript.

## Supplementary Materials

The following supporting information can be downloaded at: https://www.mdpi.com/article/10.3390/ani13101632/s1, Figure S1: Summary of lipids mapped to the lipid library and quantified; Figure S2: Individual Principal Component Analysis (PCA) analysis of turbot (A) and tiger puffer (B). Sm: *Scophthalmus maximus* (turbot); Tr: *Takifugu rubripes* (tiger puffer); C: the control group; CHO: the cholesterol-supplemented group; Figure S3: Scree plot and contribution of variables to dimension-1 and -2 in the Principal Component Analysis (PCA) presented in Figure 1; Figure S4: The crude lipid content in the muscle of turbot and tiger puffer (% wet weight, mean ± standard error). C: the control group; CHO: the cholesterol-supplemented group; Figure S5: Heatmap of quantified phosphatidic acids (PA), lysophosphatidic acid (LPA) and phosphatidylserine (PS). The average concentration of a lipid metabolite was standardized to be 0. Higher concentration than the average was labeled as orange, and lower concentration was labeled as purple. The color value as indicated in the right color bar means fold of standard deviation distant to the average concentration. Sm: *Scophthalmus maximus* (turbot); Tr: *Takifugu rubripes* (tiger puffer); C: the control group; CHO: the cholesterol-supplemented group; Figure S6: Heatmap of quantified triacylglycerol (TAG). The average concentration of a lipid metabolite was standardized to be 0. Higher concentration than the average was labeled as orange, and lower concentration was labeled as purple. The color value as indicated in the right color bar means fold of standard deviation distant to the average concentration. Sm: *Scophthalmus maximus* (turbot); Tr: *Takifugu rubripes* (tiger puffer); C: the control group; CHO: the cholesterol-supplemented group; Figure S7: Heatmap of quantified glucosylceramides (GluCer). The average concentration of a lipid metabolite was standardized to be 0. Higher concentration than the average was labeled as orange, and lower concentration was labeled as purple. The color value as indicated in the right color bar means fold of standard deviation distant to the average concentration. Sm: *Scophthalmus maximus* (turbot); Tr: *Takifugu rubripes* (tiger puffer); C: the control group; CHO: the cholesterol-supplemented group; Table S1: Formulation and proximate composition of the experimental diets (% dry matter); Table S2: Abundance of lipid classes between the cholesterol-supplemented group and the control group.
