# Supplementary material for: Dietary Cholesterol Differentially Regulates the Muscle Lipidomics of Farmed Turbot and Tiger Puffer
Source: Animals (Basel). 2023 May 13;13(10):1632. doi: 10.3390/ani13101632 (PMC10215662; doi:10.3390/ani13101632)
Supplement: Supplementary file 1 [file animals-13-01632-s001.zip › animals-2330768-supplementary.pdf]

Table S1. Formulation and proximate composition of the experimental diets (% dry matter).

Ingredients	Control	CHO
Fish meal	30	30
Corn Gluten meal	10	10
Soybean meal	10	10
Casein	10	10
Wheat meal	16.68	16.68
Brewer's yeast	10	10
Mineral premixa[a]	0.5	0.5
Vitamin premixa[a]	1	1
Monocalcium phosphate	1	1
L-ascorbyl-2-polyphosphate	0.2	0.2
Choline chlorid	0.2	0.2
Betaine	0.3	0.3
Ethoxyquin	0.02	0.02
Mold inhibitor[b]	0.1	0.1
Linseed oil	2	2
Soybean oil	1	1
Rapeseed oil	1	1
Soya lecithin	2	2
α-starch	4	3
Cholesterol[c]	0	1
Proximate composition		
Crude protein	49.75	50.14
Crude lipid	8.34	9.47
Ash	8.02	8.07

[a] Vitamin premix and mineral premix, designed for marine fish, were purchased from Qingdao Master Biotech Co., Ltd, Qingdao, China. [b] Contained 50% calcium propionic acid and 50% fumaric acid. [c] Cholesterol (AR, >95%) was purchased from Shanghai Macklin Biochemical Co., Ltd, Shanghai, China.

Table S2. Abundance of lipid classes between the cholesterol-supplemented group and the control group.

	Turbot		Tiger puffer	
**Lipid**	**parametric *P* value**	**Fold: cholesterol/control**	**parametric *P* value**	**Fold: cholesterol/control**
FFA	0.354	1.206	0.583	0.898
Acylcarnitine	0.078	2.085	0.143	1.525
DAG	0.904	0.983	0.789	0.961
TAG	0.389	1.264	0.482	1.160
PC	0.511	1.043	0.277	1.054
Plasmalogen PC	0.209	0.904	0.748	0.968
LPC	0.174	1.232	0.296	1.106
PE	0.073	0.903	0.987	1.001
Plasmalogen PE	0.117	0.863	0.351	0.932
LPE	0.789	1.060	0.355	0.812
PS	0.411	1.077	0.027	1.033
LPS	0.216	0.885	0.556	0.940
PG	0.506	1.067	0.156	0.871
BMP	0.587	0.936	0.020	2.010
PI	0.457	0.953	0.828	1.016
LPI	0.556	1.470	0.717	1.062
PA	0.243	1.127	0.243	1.237

LPA	0.667	1.071	0.071	1.315
CL	0.429	0.923	0.393	1.064
Sph	0.554	0.866	0.861	1.035
Cer	0.705	0.910	0.507	0.970
SM	0.185	0.880	0.768	1.020
GalCer	0.925	1.033	0.701	1.128
GluCer	0.529	1.255	0.195	1.415
LacCer	0.845	0.954	0.359	2.460
Gb3	0.093	0.772	0.601	0.981
GM3	0.266	1.247	0.282	1.126
SL	0.209	0.783	0.599	1.154
Cho	0.013	1.256	0.093	1.113
CE	0.000	6.230	0.069	1.547

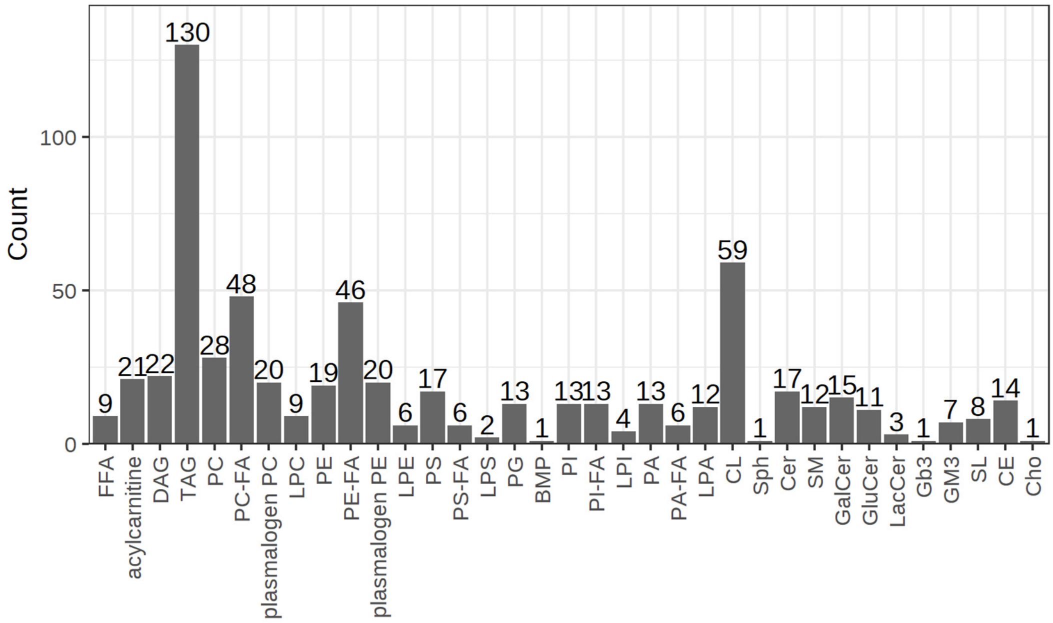

Figure S1. Summary of lipids mapped to the lipid library and quantified.

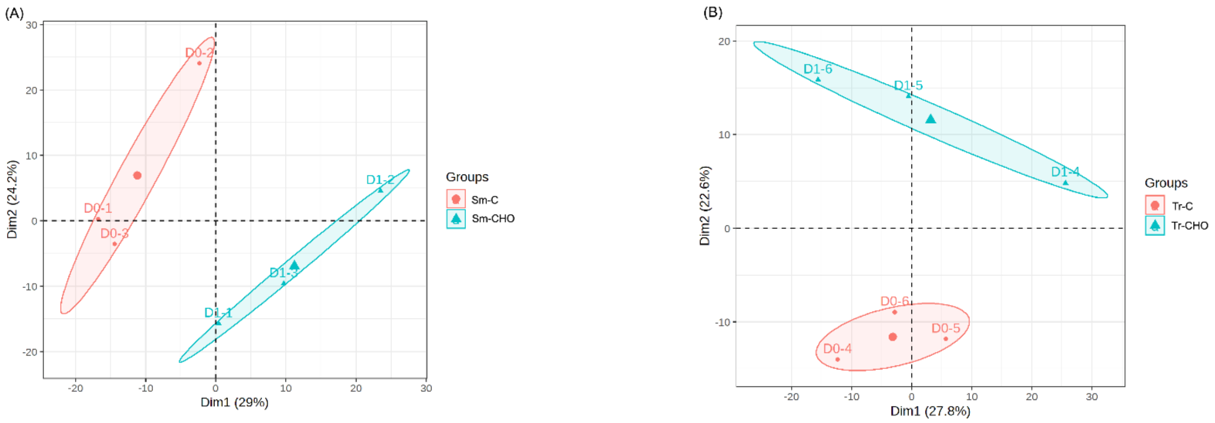

Figure S2. Individual Principal Component Analysis (PCA) analysis of turbot (A) and tiger puffer (B). Sm: *Scophthalmus maximus* (turbot); Tr: *Takifugu rubripes* (tiger puffer); C: the control group; CHO: the cholesterol-supplemented group.

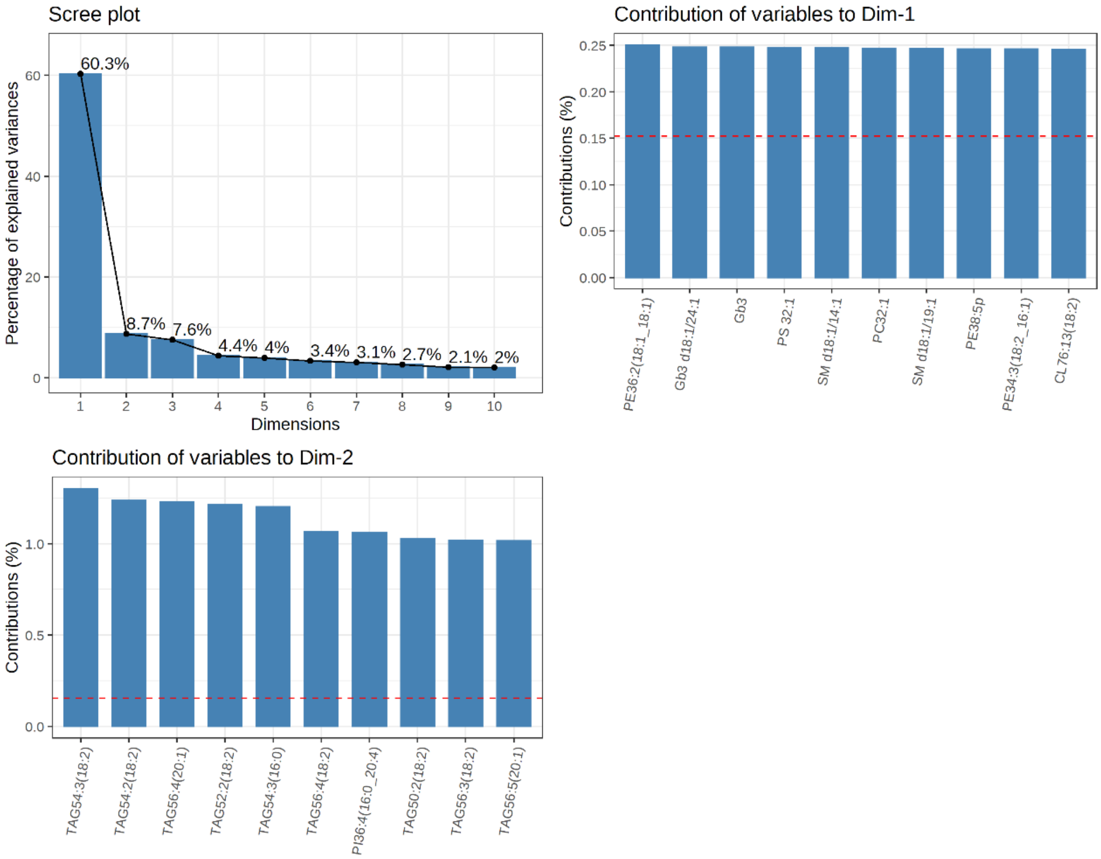

Figure S3. Scree plot and contribution of variables to dimension-1 and -2 in the Principal Component Analysis (PCA) presented in Figure 1.

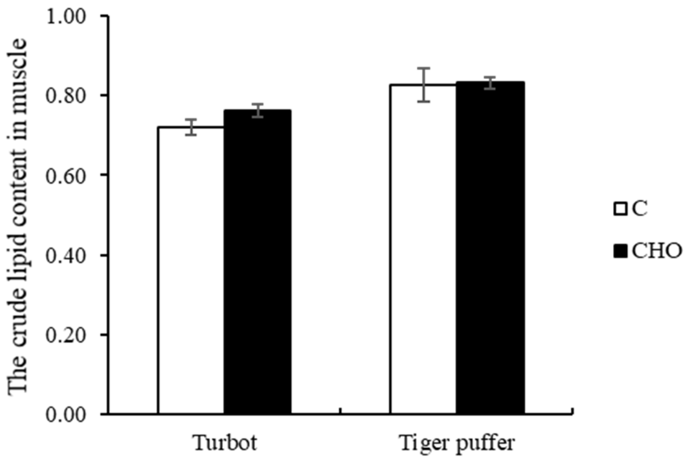

Figure S4. The crude lipid content in the muscle of turbot and tiger puffer (% wet weight, mean ± standard error). C: the control group; CHO: the cholesterol–supplemented group.

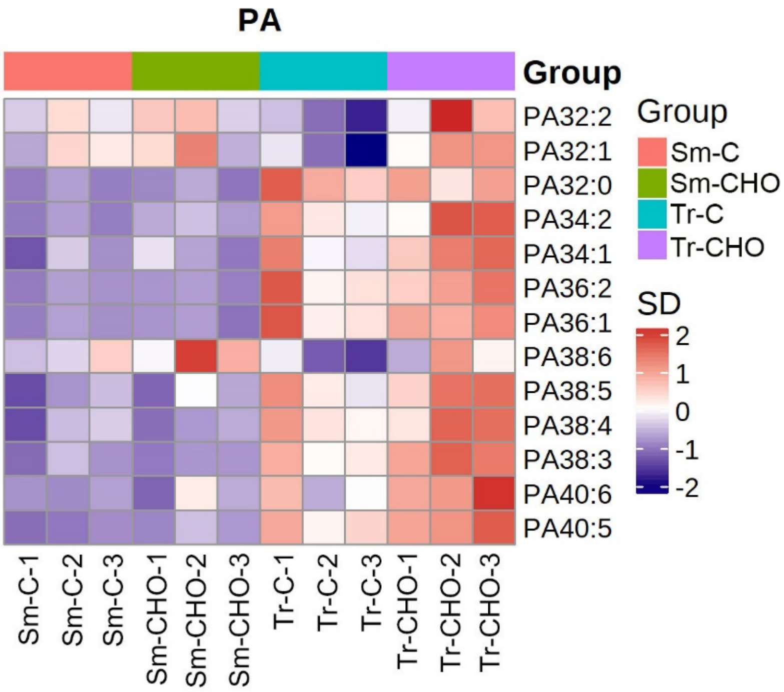
PA
Group
PA32:2
PA32:1
PA32:0
PA34:2
PA34:1
PA36:2
PA36:1
PA38:6
PA38:5
PA38:4
PA38:3
PA40:6
PA40:5
Group
Sm-C
Sm-CHO
Tr-C
Tr-CHO
SD
2
1
0
-1
-2
Sm-C-1
Sm-C-2
Sm-C-3
Sm-CHO-1
Sm-CHO-2
Sm-CHO-3
Tr-C-1
Tr-C-2
Tr-C-3
Tr-CHO-1
Tr-CHO-2
Tr-CHO-3

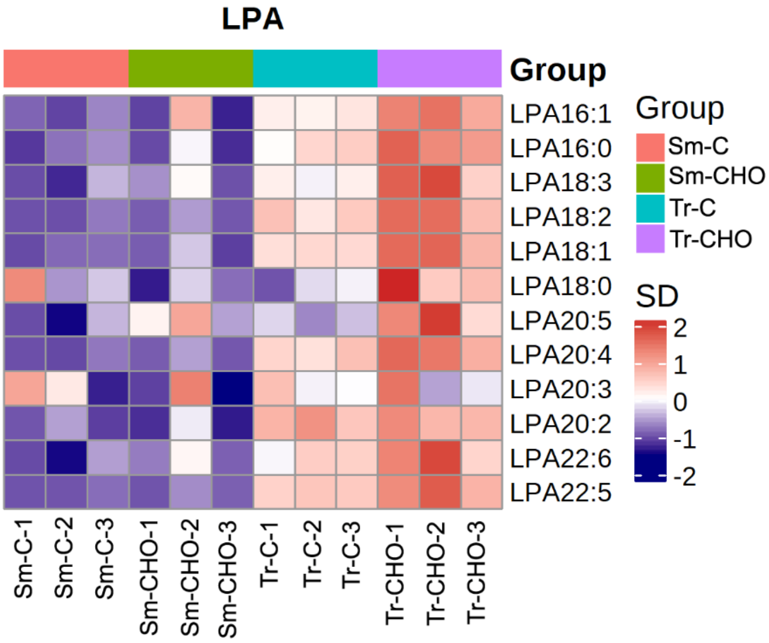
LPA
Group
LPA16:1
LPA16:0
LPA18:3
LPA18:2
LPA18:1
LPA18:0
LPA20:5
LPA20:4
LPA20:3
LPA20:2
LPA22:6
LPA22:5
Group
Sm-C
Sm-CHO
Tr-C
Tr-CHO
SD
2
1
0
-1
-2
Sm-C-1
Sm-C-2
Sm-C-3
Sm-CHO-1
Sm-CHO-2
Sm-CHO-3
Tr-C-1
Tr-C-2
Tr-C-3
Tr-CHO-1
Tr-CHO-2
Tr-CHO-3

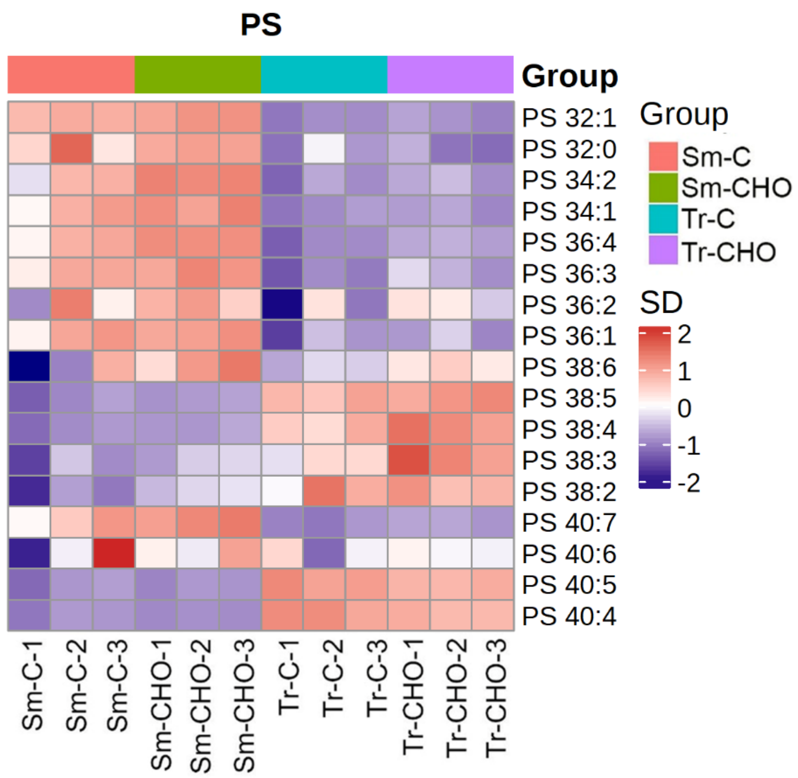

Figure S5. Heatmap of quantified phosphatidic acids (PA), lysophosphatidic acid (LPA) and phosphatidylserine (PS). The average concentration of a lipid metabolite was standardized to be 0. Higher concentration than the average was labeled as orange, and lower concentration was labeled as purple. The color value as indicated in the right color bar means fold of standard deviation distant to the average concentration. Sm: *Scophthalmus maximus* (turbot); Tr: *Takifugu rubripes* (tiger puffer); C: the control group; CHO: the cholesterol–supplemented group.

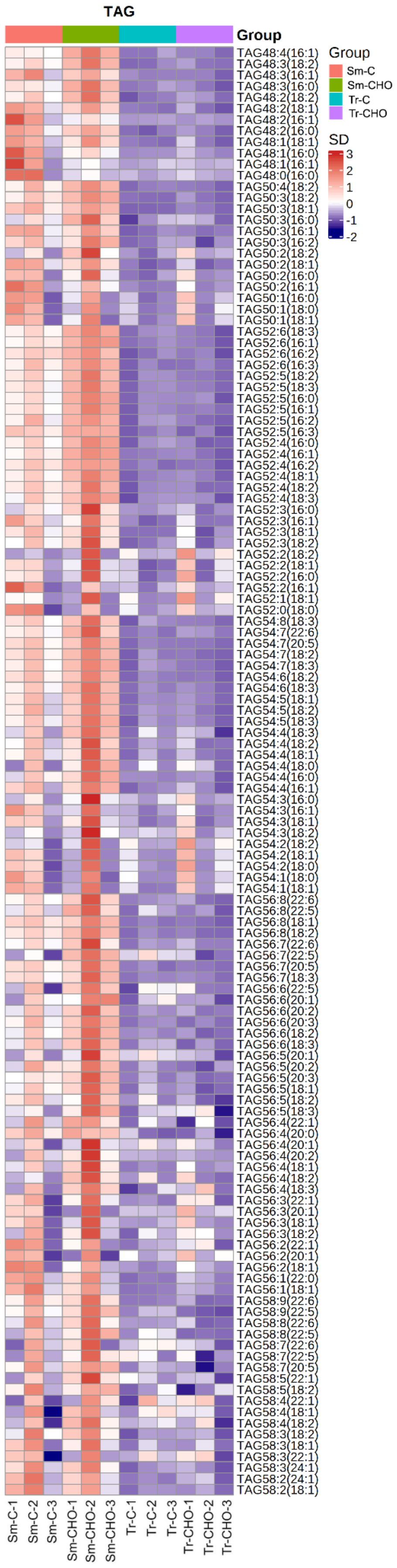

Figure S6. Heatmap of quantified triacylglycerol (TAG). The average concentration of a lipid metabolite was standardized to be 0. Higher concentration than the average was labeled as orange, and lower concentration was labeled as purple. The color value as indicated in the right color bar means

fold of standard deviation distant to the average concentration. Sm: *Scophthalmus maximus* (turbot); Tr: *Takifugu rubripes* (tiger puffer); C: the control group; CHO: the cholesterol–supplemented group.

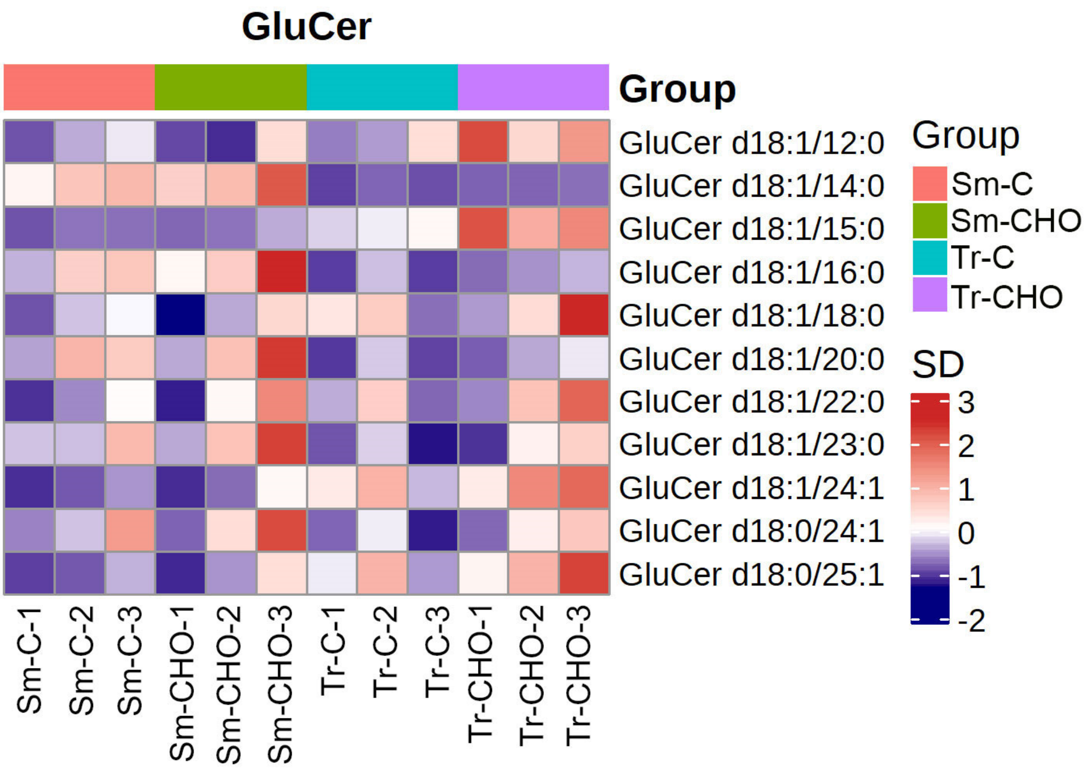

Figure S7. Heatmap of quantified glucosylceramides (GluCer). The average concentration of a lipid metabolite was standardized to be 0. Higher concentration than the average was labeled as orange, and lower concentration was labeled as purple. The color value as indicated in the right color bar means fold of standard deviation distant to the average concentration. Sm: *Scophthalmus maximus* (turbot); Tr: *Takifugu rubripes* (tiger puffer); C: the control group; CHO: the cholesterol–supplemented group.
